# Draft Genome Sequences of Mycobacterium setense Type Strain DSM-45070 and the Nonpathogenic Strain Manresensis, Isolated from the Bank of the Cardener River in Manresa, Catalonia, Spain

**DOI:** 10.1128/genomeA.01485-14

**Published:** 2015-02-05

**Authors:** Gabriel Rech, Cristina Vilaplana, Juan Velasco, Raquel Pluvinet, Sheila Santín, Cristina Prat, Esther Julián, Fernando Alcaide, Iñaki Comas, Lauro Sumoy, Pere-Joan Cardona

**Affiliations:** a Genomics and Bioinformatics Group, Institute for Predictive and Personalized Medicine of Cancer (IMPPC), Can Ruti Campus, Badalona, Catalonia, Spain; b Unitat de Tuberculosi Experimental, Institut Germans Trias i Pujol (IGTP), Can Ruti Campus, Badalona, Catalonia, Spain; c Departament de Genètica i de Microbiologia, Universitat Autònoma de Barcelona, Bellaterra, Catalonia, Spain; d Centro de Investigación Biomédica en Red en Enfermedades Respiratorias (CIBERES), Madrid, Spain; e Departament de Microbiologia, Hospital Universitari Germans Trias i Pujol, Badalona, Catalonia, Spain; f Hospital Universitari de Bellvitge-Institut de Investigació Biomèdica de Bellvitge (IDIBELL), Servei de Microbiologia, L’Hospitalet de Llobregat, Catalonia, Spain; g Departament de Patologia i Terapèutica Experimental, Universitat de Barcelona, L’Hospitalet de Llobregat, Catalonia, Spain; h Genomics and Health Unit, FISABIO Public Health, Foundation for the Promotion of Health and Biomedical Research of Valencian Region, Valencia, Spain; i Centro de Investigación Biomédica en Red en Epidemiología y Salud Pública (CIBERESP), Madrid, Spain

## Abstract

We present here the draft genome sequences of two Mycobacterium setense strains. One of them corresponds to the M. setense type strain DSM-45070, originally isolated from a patient with a posttraumatic chronic skin abscess. The other one corresponds to the nonpathogenic M. setense strain Manresensis, isolated from the Cardener River crossing Manresa, Catalonia, Spain. A comparative genomic analysis shows a smaller genome size and fewer genes in M. setense strain Manresensis relative to those of the type strain, and it shows the genome segments unique to each strain.

## GENOME ANNOUNCEMENT

In the search for nonpathogenic mycobacteria that could be used as a probiotic for tuberculosis prevention, we isolated a novel bacterial strain from the Cardener River near Manresa, Catalonia, Spain. We found its 16S rRNA sequence to be identical to that of Mycobacterium setense (1). We also observed a high similarity to M. setense in other genes commonly used in the classification of Mycobacterium species, such as *rpoB*, *rpoC*, *hsp65*, and *sodA*. Thus, we named this new strain M. setense strain Manresensis.

Virulence tests based on intravenous administration in severe immunocompromised mice (SCID) revealed that Manresensis is significantly less virulent than the Mycobacterium bovis BCG vaccine SSI, even with a dose 10 times higher. In contrast, all previously described isolates from M. setense appear to be pathogenic. The type strain DSM-45070 was first isolated from a patient in Sète, France (1), and clinical isolates have been reported in Marseille, France (74023791) (2) and Iran (HNTM46, HNTM49, and HNTM91) (3). The biochemical characterization of the Manresensis and DSM-45070^T^ strains revealed further differences between them regarding growth in glucose, gelatinase, urease, 5% NaCl tolerance, and mycolic acid patterns.

To provide insight into the genetic causes of these phenotypic differences, we sequenced the genomes of M. setense strain DSM-45070^T^ and strain Manresensis. We obtained 1,771,796 (250 nucleotides [nt] long) and 22,250,521 paired-end reads, respectively, using the Illumina SBS technology on a MiSeq sequencer. After merging the paired-end reads with FLASH (4), *de novo* assemblies were generated using SPAdes version 3.0 (5). The Manresensis genome size amounted to 6.06 Mb, split into 22 contigs, with an average depth of coverage of 788×. The DSM-45070^T^ genome was 6.26 Mb long, divided into 21 contigs, with 71× coverage. The G+C contents in both strains (66.5%) were found to be expected for Mycobacterium species. The average nucleotide identity by BLAST (ANIb) calculated with JSpecies (http://www.imedea.uib.es/jspecies) was 98.5% between the two genomes, corroborating that they belong to the same species. The closest published Mycobacterium genome based on ANIb is that of M. septicum (DSM-44393) (ANIb, 87%); however, the contigs were ordered with Mauve (6) using the Mycobacterium sp. strain MCS (accession no. NC_008146) genome assembly as reference guide, since it was the closest one (ANIb, 76.5%) with a fully complete genome. In addition to the difference of 0.2 Mb in genome size, genome annotation using the RAST engine (7) also revealed differences in the gene numbers. Manresensis and DSM-45070^T^ were predicted to have 5,716 and 5,953 protein-coding genes, respectively. We mapped sequence reads from Manresensis to the DSM-45070^T^ genome and vice versa using the Burrows-Wheeler aligner (BWA) (8) to identify the segments unique to each genome. Using this strategy, we found Manresensis to have 3.7% of its genome without homology in DSM-45070^T^ and 6.7% of the DSM-45070^T^ genome without homology in Manresensis. An analysis of the gene contents of these stretches revealed 144 genes unique to Manresensis and 373 genes unique to DSM-45070^T^, which might explain the distinct phenotypes we observed between the strains.

### Nucleotide sequence accession numbers.

The whole-genome assemblies of M. setense DSM-45070^T^ and M. setense strain Manresensis have been deposited in DDBJ/EMBL/GenBank under accession numbers JTJW00000000 and JTLZ00000000, respectively. The versions described in this paper are JTJW01000000 and JTLZ01000000, respectively.
